# Approach to Functions of *BHLHE41/DEC2* in Non-Small Lung Cancer Development

**DOI:** 10.3390/ijms241411731

**Published:** 2023-07-21

**Authors:** Tatsuhiko Furukawa, Kentaro Mimami, Toshiyuki Nagata, Masatasu Yamamoto, Masami Sato, Akihide Tanimoto

**Affiliations:** 1 Department of Pathology, Graduate School Medical and Dental Sciences, Kagoshima University, 8-35-1 Sakuragaoka, Kagoshima 890-8544, Japan; akit09@m3.kufm.kagoshima-u.ac.jp; 2 Department of Pharmacy, University of Miyazaki Hospital, 5200 Kihara Kiyotake cho, Miyazaki 889-1692, Japan; kentaro_minami@med.miyazaki-u.ac.jp; 3 Department of General Thoracic Surgery, Graduate School Medical and Dental Sciences, Kagoshima University, 8-35-1 Sakuragaoka, Kagoshima 890-8544, Japan; k3701023@kadai.jp (T.N.); masa3310@m2.kufm.kagoshima-u.ac.jp (M.S.); 4 Department of Molecular Oncology, Graduate School Medical and Dental Sciences, Kagoshima University, 8-35-1 Sakuragaoka, Kagoshima 890-8544, Japan; masatatu@m2.kufm.kagoshima-u.ac.jp

**Keywords:** BHLHE41/DEC2, cancer, circadian rhythm, differentiation

## Abstract

The circadian rhythm-related genes *BHLHE40/DEC1* and *BHLHE41/DEC2* have various functions under different cell and tissue conditions. *BHLHE41/DEC2* has been reported to be both a cancer-suppressive and an oncogenic gene during cancer development. The effects of *BHLHE41/DEC2* on differentiation have been examined using *Bhlhe41/Dec2* knockout mice and/or in vitro differentiation models, and research has been conducted using genetic analysis of tumor cells, in vitro analysis of cancer cell lines, and immunohistochemical studies of the clinical samples. We summarize some of these studies, detail several problems, and consider possible reasons for contradictory results and the needs for further research.

## 1. Introduction

BHLHE40/DEC1/STRA13/SHARP2/BHLHB2 and BHLHE41/DEC2/SHARP1/BHLHB3, which belong to the basic-helix loop helix (BHLH) protein family, function as suppressive transcription factors and are involved in circadian rhythm regulation. Both are induced by the principal circadian rhythm-related genes *CLOCK* and *BMAL1* and suppress *PER* and *CRY* expression [1]. Individuals with a variant of BHLHE41/DEC2, in which arginine replaces proline at amino acid position 384 or histidine substitutes tyrosine at position 362, exhibit the human short-sleep phenotype [2,3]. BHLHE41/DEC2 can suppress orexin, a molecule to maintain mammalian arousal, but P384R-mutated BHLHE41/DEC2 has less binding activity to the prepro-orexin promoter region and decreases the expression of orexin [4].

## 2. The Functions of BHLHE41/DEC2 in Differentiation

BHLHE41/DEC2 also plays critical roles in differentiation, mainly in subsets of cell lineages, including T-helper 2 (Th2), innate-like B lymphocytes 1 (B-1), and alveolar macrophages in vivo models, and myogenesis, adipose cell and chondrocyte lineage-committed mesenchymal cells in in vitro models [5,6,7,8,9,10,11].

*Bhlhe41/Dec2*-deficient mice lack the expression of interleukin 4 (IL-4), IL-5, IL-13, and IL-17 in vitro and in vivo in an asthma model and in response to a challenge with a parasite antigen. Th2 cells express higher level of Bhlhe41/Dec2 than Th1, Th17, inducible regulatory T cell subsets, and naïve CD4^+^ and CD8^+^ T cells. After activation of naïve CD4^+^ T cells with plate-bound anti-CD3 and anti-CD28 antibodies in the presence or absence of IL-25 for 1 to 3 days, *Bhlhe41/Dec2* mRNA expression strongly increased in response to IL-25 stimulation. Differentiation from naïve CD4^+^ T cells occurs by induction of IL-4, IL-5, and IL-13 expression through enhancement of *JunB* and *Gata3* expression [5].

Bhlhe41/Dec2 is high in B-1 cells; however, Bhlhe40/Dec1 expression is low and broad in B-lymphoid lineage cells. B-1 cells provide the first line of defense against pathogens and are divided into a major subset, B-1a, and a minor subset, B-1b, based on CD5 expression. Plasma cells derived from B-1 cells are a major source of IgM. In *Bhlhe40/Dec1* and *Bhlhe41/Dec2* double-knockout (DKO) mice, B-1a cells were severely reduced compared to their wild-type (wt) counterparts. B-1a cells from *Bhlhe40/Dec1* and *Bhlhe41/Dec2* DKO mice exhibit an abnormal cell surface phenotype and altered B-cell receptor (BCR) repertoire. The comparative studies of B-1a cells from wt and *Bhlhe40/Dec1* and *Bhlhe41/Dec2* DKO mice by RNA-seq, Chip-seq, and ATAC-seq analyses revealed that Bhlhe41/Dec2 directly repressed the expression of cell cycle regulators containing cyclin H (*Ccnh*), a cyclin-dependent kinase-like protein (*Cdkl1*), a regulatory subunit of cyclin-dependent kinases (*Cks2*), two helicases (*Hells, Recql5*), a deubiquitinase (*Usp28*), and four E2F family genes (*E2f1, E2f2, E2f7, E2f8*). Furthermore, it increased expression of IgM heavy chain (*Ighm*), inhibitors of BCR signaling (*Dusp1, Dusp2, Dusp4, and Dusp6*), and survival cytokine signaling receptor components (*Il5ra, Il3ra, Csf2rb, and Csf2rb2*). *Bhlhe41/Dec2* has crucial roles in B-1 cell differentiation through regulation of the expression of these molecules [6].

The expression of *BHLHE40/DEC1* in the red pulp, peritoneal, and alveolar macrophages, and that of *BHLHE41/DEC2* in alveolar macrophages and microglia is high. In *Bhlhe40/Dec1* and *Bhlhe41/Dec2* DKO mice, alveolar macrophages showed decreased expression of epithelial cell adhesion molecule (*Epcam*), which is a signature molecule of alveolar macrophages, and reduced proliferation, probably due to the high expression of Maf and Mafb, which are negative regulators of macrophage proliferation. Genome-wide characterization of Bhlhe40/Dec1 DNA binding suggested that Bhlhe40/Dec1 and Bhlhe41/Dec2 directly repress the expression of many specific genes of the other subsets of macrophages, including *MSR1* and *CD93* expressed by peritoneal macrophages, *Sox* and *Zfp69* expressed by microglia, and *Spic* and *VCAM1*, which are master regulator and marker of red pulp macrophage, respectively. This study indicated that Bhlhe40/Dec1 and Bhlhe41/Dec2 are key regulators of the self-renewal and identity of alveolar macrophages; however, this study lacks clear discrimination between the functions of Bhlhe40/Dec1 and Bhlhe41/Dec2 and the specific functions of Bhlhe41/Dec2 are unclear [7].

In a myogenic differentiation model of *mouse* myofibroblast C2C12 cells, endogenous Bhlhe41/Dec2 protein levels gradually decreased after the induction of differentiation, and continuous Bhlhe41/Dec2 expression suppressed differentiation by inhibiting myogenic regulatory transcription factors MyoD homodimer, E47 homodimer, and MyoD/E47 heterodimer, which thoroughly interacted with Bhlhe41/Dec2. Bhlhe41/Dec2 also decreased cyclin D1 and p21 expression [8]. Based on an in-situ hybridization study of *mouse* embryos, Bhlhe41/Dec2 expression was detectable in E10.5 and E11.5 in myotomes in a dorsoventral stripe and developing limbs in *mouse* embryos, and thus, it is unlikely that Bhlhe41/Dec2 suppresses the initiation of myogenesis that is regulated by another BHLH family gene, myogenin (E8.5) and myogenic regulator factor 4 (E9.5) [12]. BHLHE41/DEC2 has been reported to have a myogenesis-inhibitory function in a human muscle disease. Inclusion body myositis (IBM) is a slowly progressive disease of unknown etiology, characterized by asymmetric muscle weakness. Most patients with IBM eventually develop severe motor impairments, including walking difficulties. Satellite cell-dependent muscle regeneration occurs in IBM; however, the regenerated muscles do not reach the point where sufficient strength can be exerted. Mesoangioblasts are stem cells associated with blood vessels that have the potential to differentiate into a variety of mesoderm-derived cells, including skeletal, cardiac, and smooth muscle cells via differentiation-induction conditions. Mesoangioblasts isolated from patients with IBM highly express BHLHE41/DEC2 and lack sufficient potency for myogenic differentiation. Silencing *BHLHE41/DEC2* could rescue mesoangioblasts from myogenic defect and induce them to differentiate into multinucleated myosin-positive myotubes. This study indicates that BHLHE41/DEC2 is a promising therapeutic target for IBM management [9].

Adipose cell differentiation is induced by the transient expression of C/EBPβ and C/EBPδ, followed by a self-reinforcing loop between C/EBPα and peroxisome proliferator-activated receptor γ (PPARγ). C/EBPα directly binds to the *PPARγ* promoter and PPARγ also induces *C/EBPα* expression. In the adipose cell differentiation model of 3T3L1 *mouse* fibroblast cell, Bhlhe41/Dec2 could retain histone deacetylase1 (HDAC1) and the histone methyltransferase G9a in the C/EBPα and *PPARγ* promoter regions to disrupt this loop and suppress the differentiation [10].

During chondrogenic differentiation of *human* mesenchymal stem cells (MSCs), *BHLHE41/DEC2* mRNA expression increases transiently. Overexpression of *BHLHE41/DEC2* does not inhibit cell proliferation but inhibits an increase in DNA content, and expression of several chondrocyte-related genes, including aggrecan and type X collagen α1, potentially through attenuation of fibroblast growth factor 18 (Fgf18), which is involved in the proliferation and differentiation of chondrocytes [13]. In addition, BHLHE41/DEC2 decreases cyclin D1 and increases p16^INK4^ and p21. These data suggest that BHLHE41/DEC2 suppresses the extent of terminal differentiation of chondrocytes, although the direct function of BHLHE41/DEC2 and its biological meaning are still unclear [11]. In myogenic differentiation of C2C12 cells, BHLHE41/DEC2 can decrease p21 expression, because BHLHE41/DEC2 suppresses MyoD, which induces p21 expression [12]. Conversely, in *human* MSCs, BHLHE41/DEC2 expression tends to induce p21 expression, although this change is not significant. BHLHE41/DEC2 has an additional suppressive effect on FGF18 expression and p21 was assumed to be suppressed under the FGF18-FGFR3-STAT1-p21 cascade [13]. However, there is no clear explanation for this discrepancy in this report [11]. There is a possibility of moderate stabilization of TP53 by BHLHE41/DEC2 expression, although there is no clear evidence. These effects of BHLHE41/DEC2 on differentiation are summarized in Table 1.

## 3. Oncogenic and Tumor-Suppressive Functions of BHLHE40/DEC1 and BHLHE41/DEC2

This sounds paradoxical; however, *BHLHE41/DEC2* is a tumor-suppressive and oncogenic molecule [14,15]. BHLHE41/DEC2 has been reported to act as a tumor suppressor in several types of cancers. Its function is pivotal, especially in triple-negative breast cancers (TNBCs). TNBCs lack the expression of estrogen receptors (ER), progesterone receptors, and *human* epidermal growth factor receptor-2, and have highly aggressive characteristics; therefore, patients with TNBCs have poorer prognoses than those with other types of breast cancer. Hypoxia-inducible factor (HIF)-1 expression is high in TNBC cells, which can induce invasion, metastasis, and chemotherapy resistance, and is associated with unfavorable prognoses in patients with TNBC [16]. Both *BHLHE40/DEC1* and *BHLHE41/DEC2* expressions are induced by HIF-1 stabilization under hypoxic conditions [17]. In TNBC cells, BHLHE41/DEC2 can bind and inhibit HIF-1α and HIF-2α functions by promoting their proteasomal degradation that is independent of the von Hippel–Lindau tumor suppressor, which is an E3 ligase of HIF-1α and HIF-2α proteins, and suppress tumor invasion and metastasis in an in vivo model [18]. In addition to HIF-1, the expression of X-Box Binding Protein 1 (XBP1), an endoplasmic reticulum stress-regulating transcription factor, also increases and plays a crucial role in TNBC cells, because XBP1 supports tumor stem cell proliferation in TNBC. Based on genome-wide mapping of the XBP1 transcriptional regulatory network, the XBP1 and HIF-1 assembly transcriptional complex recruits RNA polymerase II to HIF-1-target genes containing Vascular Endothelial Growth Factor A (*VEGFA*)*,* Pyruvate Dehydrogenase Kinase 1 (*PDK1*)*, GLUT1/SLC2A1* and DNA Damage Inducible Transcript 4 *(DDIT4)* and enhances these genes even under normoxic conditions [19]. In TNBC, BHLHE41/DEC2 is a crucial tumor-suppressing molecule, since it can inhibit HIF-1 and possibly XBP1 functions and cancer stem cells. Also, in *mouse* fibroblast NIH3T3 cells and *mouse* sarcoma 180 cells, under hypoxic conditions, BHLHE41/DEC2 interacted with HIF-1α and decreased the binding of HIF-1α to the hypoxia response element in the *VEGF* promoter [20]. BHLHE41/DEC2 also suppresses the growth of thyroid cancer cell lines and their expression of HIF-1α [21]. These data suggest that BHLHE41/DEC2 plays a crucial role in suppressing HIF-1 functions. 

**Figure 1:**
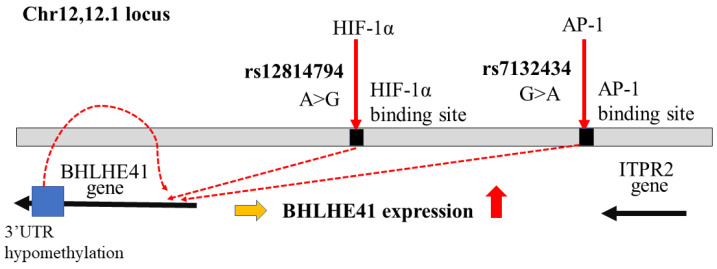
Genetic and epigenetic changes on chromosome 12 relating to *BHLHE41/DEC2* expression in ccRCC development. *BHLHE41/DEC2* expression via SNP rs7132434, rs12814794, and hypomethylation of *BHLHE41/DEC2*-3′UTR in ccRCC development. The red arrows indicate the transcription factors can bind around the SNPs. The red dashed lines show to induce expression of BHLHE41/DEC2 by these changes.

In contrast, several studies support that BHLHE41/DEC2 has the functions in the development of renal cell cancer (RCC). Stage-specific activation of HIF-1α and HIF-2α plays an essential role in RCC development [22,23]. The fact that HIF plays a critical role in both TNBC and RCC development is not coincidental, although the effects of BHLHE41/DEC2 on TNBC and RCC appear to be opposed. The alignment of polymorphisms related to RCC susceptibility and HIF-1 binding sites was observed in a genome-wide association study using chromatin immunoprecipitation sequencing (CHIP-Seq), in which it was found that rs12814794 single nucleotide polymorphisms (SNP) at chr 12p12.1 were related to RCC susceptibility. A chromatin conformation assay, Capture-C, identified interaction with the polymorphic HIF binding site at chr 12p12.1 and the promoter of the *BHLHE41/DEC2* gene. RCC is derived from renal tubular epithelial cells which express HIF-1α. The change from A to G rs12814794 can create a new HIF-1α binding site and enhance *BHLHE41/DEC2* expression in normal *human* primary renal tubular cells in the presence of the HIF-1α protein [24] (Figure 1). This genetic change can induce BHLHE41/DEC2 expression during cancer development under HIF-1α expression; however, in this study the direct effects of BHLHE41/DEC2 were not examined. In another study, clear cell RCC (ccRCC) development was strongly associated with rs7132434. This SNP could become an additional AP-1 binding site and induce BHLHE41/DEC2 expression, which, in turn, induces IL-11, but not HIF-1α expression in RCC cells (Figure 1). Genomic database analysis did not reveal any relationship between *BHLHE41/DEC2* mRNA expression and adverse pathogenic factors in clinical RCC samples or the prognosis of patients with RCC. However, in a xenograft model with ACHN cells, but not in-vitro models of several RCC cell lines, BHLHE41/DEC2 promoted cell growth without HIF-1 expression [15]. A specific increase in BHLHE41/DEC2 protein expression in ccRCC was observed in an immunohistochemical study [25]. These data suggest that BHLHE41/DEC2 directly affects tumor development without HIF. In contrast, in another study, *BHLHE41/DEC2* expression increased, possibly due to DNA hypomethylation in the 3′ untranslated region of *BHLHE41/DEC2* in *human* ccRCC cells (Figure 1). The knockdown of *BHLHE41/DEC2* in A498 and CAKI-1 RCC cell lines reduced cell proliferation and migration with attenuation of phosphorylation of p70S6kinase and increased E-cadherin expression. The results of this study also indicated an increase in *BHLHE41/DEC2* expression using quantitative PCR of 50 clinical samples and immunoblotting of five pairs of RCC-positive and adjacent normal kidneys [26]. This report implies that BHLHE41/DEC2 induces the activation of the mTOR cascade and promotes epithelial-mesenchymal transition (EMT). The authors assumed that BHLHE41/DEC2 can positively regulate EMT transcription factors; however, this result is inconsistent with other reports on epithelial cancer cells as described later. 

There are some discrepancies between the studies by Bigot and Shen, which may be due to differences in the experimental conditions [15,26]. For example, in the study by Bigot, BHLHE41/DEC2 expression was introduced into RCC cell lines without BHLHE41/DEC2 expression. In the study by Shen, the authors used BHLHE41/DEC2-expressing cells and knockdown of *BHLHE41/DEC2*. Cells lacking endogenous BHLHE41/DEC2 expression may lack other proteins that interact with BHLHE41/DEC2 and induce in vitro proliferation and migration. However, neither study indicated that BHLHE41/DEC2 expression is related to pathological grade or the overall survival rate in patients with ccRCC from TCGA database analysis. These results suggest that BHLHE41/DEC2 might be involved in the development of ccRCC, but is not strongly associated with cancer progression. This hypothesis is compatible with HIF-1α protein expression, only in an early phase of RCC development; some RCC cells lack expression of HIF-1α and express HIF-2α. BHLHE41/DEC2 might have a critical function in cancer development but may not always be required for maintaining advanced tumors. It might, however, be useful as a stratifying marker gene of early diagnosis. Although the oncogenic functions of BHLHE41/DEC2 and its relationship with HIF in RCC have not yet been completely elucidated, all reports indicate that BHLHE41/DEC2 is involved in the development and progression of renal cell cancer development. Based on analyses of the functions of BHLHE41/DEC2 in TNBC and ccRCC, it is expected that BHLHE41/DEC2 interacts with HIF-1 to either activate or suppress the hypoxia response element, possibly dependent on additional partner molecules. HIFs are also activated by *BHLHE41/DEC2*, as Bhlhe41/Dec2 has been reported to enhance *JunB* and *Gata3* expression during the differentiation of Th2 cells [5].

In addition to RCC, BHLHE41/DEC2 promotes the development of a type of acute myelogenous leukemia (AML) with mixed lineage leukemia (*MLL*) gene rearrangement by the t(6;11) (q27;p23) translocation, named *MLL-AF6*. The *MLL* gene at 11q23 encodes a nuclear protein with multiple functional domains, many of which can bind to several proteins containing PSIP1/LEDGF and MEN1, and regulate epigenetically defined developmental genes. During hematopoiesis, MLL activates *HOXC8*, *HOXC6*, and *HOXA9*. *MLL* has been reported to fuse with more than 80 partner genes containing six common partner genes. The common partner genes are ALL1-fused gene chromosome *(AF) 4*, *AF6*, *AF9*, *AF10*, eleven-nineteen leukemia (*ENL*), and elongation factor for RNA polymerase II (*ELL*) [27]. *MLL* rearrangements are detected in 80% of infant acute leukemia cases, approximately 15% of children with AML, and 10–15% of adults with chemotherapy-related leukemia. *AF6/AFDN*, also known as afadin, is a cytoplasmic and nuclear protein with a single PDZ and two RAS-association domains. MLL-fusion proteins, including *MLL-AF6*, can interact with DOT1L, an H3K79 methyltransferase, and link the di- or tri-methylation of H3K79 to MLL-AF6 target genes. DOT1L is indispensable to sustaining *MLL-AF6* leukemia cells [28]. In addition, MLL-AF6 sequesters AF6 from the cytoplasm to the nucleus and triggers RAS activation. RAS activation in MLL-AF6 AML may explain the poorer prognosis of the patients with *MLL-AF6* leukemia [29]. *BHLHE41/DEC2* is specifically overexpressed in *MLL-AF6* AML cells. BHLHE41/DEC2 interacts with the oncogenic chimeric fusion protein MLL-AF6, which activates chromatin abnormalities by interacting with Dot1L. MLL-AF6 and DOT1L directly upregulates *BHLHE41/DEC2* expression in *MLL-AF6* AML cells. Suppression of *BHLHE41/DEC2* expression induces apoptosis in *human MLL-AF6* AML cells. *MLL-AF6*-expressing hematopoietic stem cells derived from mice with a genetic deletion of *Bhlhe41/Dec2* delay leukemia development and decrease the potential for leukemia initiation. Mechanistically, BHLHE41/DEC2 binding sites in *MLL-AF6* leukemia cells were enriched with H3K4me3 and H3K27ac transcriptionally active markers across the genome and activates genes related to the cell cycle, TGF-β signaling, FoxO signaling, HIF-1 signaling, and cancer [30].

*Cyclin D1* is thought to be a target of BHLHE41/DEC2 during myogenic and chondrogenic differentiation, as described above. In *human* mammary epithelial (HME) cells, BHLHE41/DEC2 and bexarotene, which is a vitamin A analog that induces BHLHE41/DEC2, suppress cyclin D1 expression and the cell growth, but not in ER-positive breast cancer MCF-7 cells [31]. In contrast, overexpression of BHLHE41/DEC2, but not BHLHE40/DEC1, increased the proliferation of MCF-7 under normoxic and hypoxic conditions, via AKT phosphorylation and *c-MYC* expression [32]. In glioblastoma U87 and U251 cells, BHLHE41/DEC2 overexpression induces ERK phosphorylation and cyclin D1 and *D3* expression [33]. Thus, the functions of BHLHE41/DEC2 in proliferation are not always consistent among reports, possibly because we do not have sufficient information regarding the interacting proteins under specific conditions, which vary depending on the background of the cells.

However, the EMT appears to be more consistent. The EMT frequently contributes to cancer cell invasion and metastasis, and worsens prognosis of the patients with cancers [34,35]. In *human* pancreatic cancer BxPC-3 cells, the knockdown of BHLHE41/DEC2 increased the nuclear expression of an EMT transcription factor, *SNAI2*, in the presence of TGF-β. BHLHE41/DEC2 inhibited EMT by regulating *SNAI2* [36]. The colon cancer cell lines HCT116 and Lovo showed lower expression of *BHLHE41/DEC2* than normal colon epithelial cells. These cells increased migration and proliferation under hypoxic conditions, with increasing HIF-1α, N-cadherin, vimentin, and MMP9 but reduced epithelial marker protein E-cadherin, while BHLHE41/DEC2 alleviated the EMT cascade protein and increased E-cadherin expression under hypoxic conditions. BHLHE41/DEC2 expression also suppressed HCT116 xenograft tumor growth [37]. Comparison of the mRNA levels of *BHLHE41/DEC2* between 20 normal endometrial tissue specimens (NEM) and 37 primary *huma* endometrial cancer (HEC) specimens showed that HEC had a significantly higher expression of BHLHE41/DEC2. The mRNA levels of *BHLHE40/DEC1* and *BHLHE41/DEC2* were significantly higher in tumors at stage IA than in those at stage IB. Expressions of *BHLHE41/DEC2* and *TWIST1* were inversely correlated with each other. In an immunohistochemical study of surgical samples from 86 clinical HEC cells, BHLHE40/DEC1 and BHLHE41/DEC2 expression was limited to non-invasive samples. BHLHE40/DEC1 and BHLHE41/DEC2 suppress the expression of the EMT transcription factors, *SNAI1*, *SNAI2*, and *TWIST1*. In particular, during *TWIST1* transcription, both BHLHE40/DEC1 and BHLHE41/DEC2 compete with SP1 for DNA binding, leading to reduced TWIST1 transcription [38]. These studies suggest that BHLHE41/DEC2 inhibits EMT transcription factors.

## 4. BHLHE41/DEC2 as a Tumor Suppressor Protein in NSCLC Development

According to recent statistical data, cancer-related mortality rates in the USA have been declining due to a steady decrease in incidence, likely due to the decreasing number of smokers, and the progression of molecular targeted therapies and immune checkpoint inhibitors. Nevertheless, lung cancer remains the leading cause of cancer-related deaths (21%) [39]. Several oncogenes and tumor suppressor genes have been identified as molecules associated with the development of non-small cell lung cancer (NSCLC). However, similar to the multistep model of colorectal cancer, the stages of cancer development in NSCLC remain unknown. Therefore, it is critical to understand the developmental processes in lung cancer to identify further therapeutic targets. Previously, BHLHE41/DEC2 has been reported to function as a tumor suppressor by downregulation of cyclin D in NSCLS [14]. We found that BHLHE41/DEC2 plays a crucial role in NSCLC development and hypothesized that the loss of BHLHE41/DEC2 expression may be an early step in the development of NSCLC. BHLHE41/DEC2 expression is associated with better prognosis in patients with lung adenocarcinoma (LUAD). Induction of BHLHE41/DEC2 expression resulted in autophagic cell death in *huma* lung cancer cells [40]. The Cancer Genome Atlas data, cBioPortal, provides information on genetic changes containing gene amplification, truncated mutation of BHLHE41/DEC2 in lung squamous cancer (LUSC), and data on amplification, point mutation, and *SHROOM2-BHLHE41* gene fusion in LUAD; however, there are no data on mutation in small cell lung cancer (SCLC). This might reflect the difference in cancer development background between NSCLC and SCLC, although the meaning of these genetic changes of BHLHE41/DEC2 is still unclear. Immunohistochemical studies showed that BHLHE41/DEC2 expression is almost exclusively limited to the lepidic growth part of LUAD, in situ adenocarcinoma, very early LUSC cells, and normal lung epithelial cells. Our observations indicated that most surgically resected LUSC samples lost BHLHE41/DEC2 expression. In addition, early LUSC can be effectively removed using radiofrequency ablation. Therefore, it is difficult to obtain information regarding BHLHE41/DEC2 function in LUSC. BHLHE41/DEC2 is expected to be an early inactivated molecule in NSCLC, possibly because BHLHE41/DEC2 is vulnerable to protein stability and epigenetic regulation of mRNA expression. Identifying partner molecules is expected to be an important step in understanding the functions of BHLHE41/DEC2 in NSCLC development. Clearly, reproducible models of cancer development are required.

## 5. Post-Translational Modifications Regulate the Functions of BHLHE40/DEC1 and BHLHE41/DEC2

SUMOylation is a post-translational modification that regulates several important cellular functions. In the SUMOylation process, a small ubiquitin-like modifier (SUMO) protein is covalently attached to a lysine residue in a consensus sequence, by enzymes consistent with E1-activating enzyme (AOS1/UBA2), E2-conjugating enzyme (UBC9), and sometimes E3 ligases, RAnBP2 and PIAS. In contrast, SUMOylation is negatively regulated by deSUMOylation with sentrin-specific protease (SENP) proteins, which are SUMO-specific isopeptidases comprising six cysteine proteases. Under hypoxic conditions, the activities of SENP1 and SENP3 were fully and reversibly suppressed, and SUMOylation was enhanced. SUMOylation can be recognized as another mechanism of adaptation to hypoxic conditions, rather than HIF-1 stabilization. From searches of hypoxia-induced SUMO1 targeting proteins using comparative mass spectrometry of HeLa cell extract, 48 SUMOylation proteins were defined, with more than twice as many in hypoxia than in normoxia. These proteins include SUMO ligases, RanBP2 and PIAS2, glucose transporter 1, several transcriptional regulators, and chromatin regulators. BHLHE40/DEC1 was identified as one of the more than five-fold SUMOylated target proteins belonging to a subgroup, which is composed of the other transcriptional repressors, *FSBP*, *NAB1*, *KCTD1*, *KCTD15*, or *ETV6*. Expression of *PGC-1α*, a master regulator of metabolism, was more strongly suppressed in wt BHLHE40/DEC1 than in the SUMOylated lysine-deficient mutant BHLHE40 under hypoxia [41].

Starvation conditions increased SUMOylation of BHLHE40/DEC1 at two major SUMOylation sites, K159 and K279, in MCF-7 cells, and SENP1 reduced SUMOylation. SUMOylation of BHLHE40/DEC1 promotes the repression of CLOCK/BMAL1-heterodimer-mediated transcriptional activity by interacting with HDAC1. The authors’ results also suggested that SUMOylation of BHLHE40/DEC1 inhibits ubiquitination and ubiquitin-proteasome degradation [42]. BHLHE40/DEC1 overexpression suppresses the proliferation of NIH3T3 *mouse* fibroblast cells and embryonic fibroblasts from *Bhlhe40/Dec1* knockout mice via SUMOylation of BHLHE40/DEC1. SUMOylation of BHLHE40/DEC1 enhances its interaction with HDAC1. In turn, HDAC1 decreases the SUMOylation of BHLHE40/DEC1 and attenuates the cyclin D1 suppressive effect of BHLHE40/DEC1 [43]. One observation from this study, that HDAC1 expression suppressed the attenuation effect of BHLHE40/DEC1 on cyclin D1, is inconsistent with results from other studies. Further studies are needed to clarify the biological effects of SUMOylation of Bhlhe40/Dec1 and exogenous HDAC1.

The *mouse* Bhlhe41/Dec2 protein has two SUMOylation consensus sequences, OQKLE and IKQE, containing SUMOylation sites K240 and K255, as does Bhlhe40/Dec1. In the C2C12 myogenesis model, Bhlhe41/Dec2 suppressed terminal differentiation. SUMOylation of Bhlhe41/Dec2 enhances the recruitment of the corepressor G9a and histone H3 lysine 9 demethylations (H3K9me2) to the *MyoD* promoter. Mutant Bhlhe41/Dec2, with arginine instead of lysine at positions 240 and 255, decreased the suppressive function, and SENP1 almost abolished the suppression of myogenesis by Bhlhe41/Dec2 [44]. Also, in 3T3L1 cells, induction of adipose cell differentiation could increase SENP1 expression and coincide with attenuation of SUMOylation of Bhleh41/Dec2. This observation is consistent with the deSUMOylation of Bhleh41/Dec2 upon Senp1 expression. Compared to Senp1-expressing *mouse* embryonic fibroblasts, embryonic fibroblasts derived from *Senp1* knockout mice with adipose cell induction had lower *RRARγ* promoter activity, with low expression of its target genes including adipocyte Protein 2 *(aP2),* adiponectin, and lipoprotein lipase *(Lpl)*, which increase in the differentiated adipocyte. Mutant Bhleh41/Dec2, without the main SUMOylation lysine residues had lower suppressor activity of *RRARγ* promoter [45]. These observations of SUMOylation and deSUMoylation demonstrate how interacting with other proteins has crucial effects on the functions of BHLHE40/DEC1 and BHLHE41/DEC2.

BHLHE40/DEC1 stability is controlled through SCFβTrCP, which mediates the ubiquitin-proteasome system dependent on the phosphorylation of BHLHE40/DEC1 by casein kinase I. BHLHE40/DEC1 protein increases by suppressing ubiquitination in an ATM/ATR-dependent manner by USP17 ubiquitin protease, after exposure to anticancer agents, etoposide or doxorubicin, in *huma* osteosarcoma U2OS cells and *huma* colon cancer HCT116 cells, both of which have wt *TP53* [46]. BHLHE41/DEC2 may be similarly regulated because they have similar casein kinase 1 consensus sequences. Therefore, the stability of BHLHE41/DEC2 may be regulated by the ubiquitin-proteasome system.

To understand the precise role of BHLHE41/DEC2 in a specific context, it is necessary to interpret its effects carefully. Therefore, it is necessary to identify the interacting proteins that suppress gene expression to suppress cancer development.

## 6. Discussion

Our previous study has three main limitations [40]. First, the number of early LUSQ samples was limited. It is difficult to detect early LUSQ on chest radiographs, and even if detected, early lesions may be radiologically resected. Therefore, a long-term systematic collection of specimens for understanding LUSQ development is essential. Second, it is unclear how BHLHE41/DEC2 can induce autophagic cell death in early cancer cells. Finally, it is unclear how BHLHE41/DEC2 discriminates between cancer and normal cells, that is why autophagic cell death happens only in cancer cells. Reproducible models of oncogenesis are needed to answer the other two questions.

So far, genetic changes of BHLHE41/DEC2 have not been studied well; however, GEPIA (http://gepia.cancer-pku.cn/) (accessed on 6 July 2023) indicates high expression of BHLHE41/DEC2 in ovarian serous adenocarcinoma (OV), stomach adenocarcinoma (STAD), thyroid cancer, ccRCC, and so on. In addition, cBioPortal indicates genetic changes include gene amplification, missense mutation, deep deletion of *BHLHE41/DEC2*, and fusion genes *ABHD17C-BHLHE41* in STAD, *ITPR2-BHLHE41* in OV, and *BHLHE41-RERGL* in low-grade glioma. In FusionGDB (https://compbio.uth.edu/FusionGDB2/index.html) (accessed on 6 July 2023), an additional four fusion genes are registered, as *BHLHE41-NOMO1, IGL-BHLHE41, KHSRP-BHLHE41*, and *SEL1L3-BHLHE41*, although it is not known if they have these functions. The accumulation of genetic changes in several types of cancer suggests that these changes are associated with some specific process of cancer development

In conclusion, we have summarized the reasons for the complexity of BHLHE41/DEC2 functions. BHLHE41/DEC2 primarily functions as a suppressive transactivating factor that binds to E-boxes of genes. E-box is one of the most common sequences in *huma* genes. More than 30 BHLH proteins bind to E-boxes [47]. Both BHLHE40/DEC1 and BHLHE41/DEC2 are induced by the same proteins, including BMAL1/CLOCK, HIF-1, and TNFα [48]. BHLHE41/DEC2 suppresses transcription in different ways [49]. Additionally, BHLHE40/DEC1 and BHLHE41/DEC2 suppress each other [50], miR-16 can bind *BHLH41/DEC2* mRNA and inhibit expression [51], epigenetic change of RNA and N6-methyladenosine can promote BHLHE41/DEC2 translation [52], and their protein expression is regulated by post-translational modifications [43]. Therefore, BHLHE41/DEC2 could induce protein degradation [18], as BHLHE41/DEC2 also works as a transcription inducer [5,30]. In addition, we could not detect BHLHE41/DEC2 expression in most of the LUSQ samples. However, the transcriptional database of LUSQ indicates some BHLHE41/DEC2 expression in advanced LUSQ, and we carefully considered mRNA expression data in these databases. The functions of BHLHE40/DEC1 and BHLHE41/DEC2 and their interactions with other proteins are likely complex. However, we are confident that our approach will lead to significant advances in the analysis of NSCLC and BHLH transcription factors.

## Figures and Tables

**Table 1:** Summary of effects on differentiation of BHLHE41/DEC2.

Phenotype	Cells	Spices	Model	Effect	Gegens Which Receive Direct and/or Indirect Effects	REF
Th2 differentiation promotion	Th2	*mouse*	*Bhlhe41* knockout mouse	induce	JunB, Gata3	[5]
B-1a cell differentiation promotion	B-1a	*mouse*	*Bhlhe41* knockout mouse	suppress	Ccnh, Cdkl1, Cks2, Usp28 et al.	[6]
				induce	Ighm, Dusp1, cytokine signal receptor component et al.	
alveolar macrophage differentiation promotion	Alveolar macrophages	*mouse*	*Bhlhe40* and *Bhlhe41* double knockout mice	suppress	specific genes of other subtypes of macrophage	[7]
				induce	Epcam	
myogenesis inhibition	C2C12	*mouse*	in vitro induction	suppress	MyoD, E47	[8]
myogenesis inhibition	Mesoangioblasts of inclusion body myositis	*human*	in vitro induction	suppress	MyoD	[9]
adipose cell differentiation inhibition	3T3L1	*mouse*	in vitro induction	suppress	C/EBPα, PPAR γ	[10]
chondrogenic differentiation inhibition	human mesenchymal stem cells	*human*	in vitro induction	suppress	Cyclin D1, several chondrocyte-related genes possibly thorough Fgf18	[11]
				induce	p16^INK4^, p21	
